# Validation and implementation of a patient-reported experience measure for patients with rheumatoid arthritis and spondyloarthritis in the Netherlands

**DOI:** 10.1007/s10067-020-05076-6

**Published:** 2020-04-21

**Authors:** Esther Beckers, Casper Webers, Annelies Boonen, Peter M. ten Klooster, Harald E. Vonkeman, Astrid van Tubergen

**Affiliations:** 1 Department of Internal Medicine, Division of Rheumatology, Maastricht University Medical Center, P. Debyelaan 25, 6229 HX, Maastricht, The Netherlands; 2 Care and Public Health Research Institute (CAPHRI), Maastricht University, Maastricht, Netherlands; 3 Department of Psychology, Health & Technology, University of Twente, Enschede, Netherlands; 4 Department of Rheumatology, Medisch Spectrum Twente, Enschede, Netherlands

**Keywords:** Epidemiology, Outcome research, Patient perspective, Rheumatoid arthritis, Spondyloarthritis

## Abstract

**Objectives:**

To test the psychometric properties of the United Kingdom’s Commissioning for Quality in Rheumatoid Arthritis Patient-Reported Experience Measure (CQRA-PREM) in patients with spondyloarthritis (SpA) and rheumatoid arthritis (RA) and to implement this questionnaire in daily practice in the Netherlands.

**Methods:**

After a forward-backward translation procedure into Dutch, the CQRA-PREM was tested into two quality registries in daily practice. Face validity was assessed with focus group interviews. Feasibility was evaluated through completion times and interpretability of domain scores through floor and ceiling effects. Internal consistency (Cronbach’s α coefficients) and homogeneity (corrected item-total correlations) were determined. Divergent validity was assessed by Spearman’s rank correlation coefficients (*r*_s_) between the average scores of domains and outcome measures. The CQRA-PREM was implemented in daily practice, and the results were used in quality improvement cycles.

**Results:**

Face validity of the CQRA-PREM was good. The CQRA-PREM was completed by 282 patients with SpA and 376 with RA. Median time to complete the CQRA-PREM was 4.7 min. Ceiling effects were found in three out of seven domains. Internal consistency of nearly all domains was considered good (0.65 ≤ α ≤ 0.95). Thresholds for homogeneity were exceeded within three domains (*r*_p_ > 0.7), suggesting item redundancy. Divergent validity showed that nearly all domains of the CQRA-PREM were at most weakly correlated with outcomes measures (− 0.3 ≤ *r*_s_ ≤ 0.3). The CQRA-PREM could identify areas of improvement for providing patient-centered care.

**Conclusion:**

The CQRA-PREM has acceptable psychometric properties and has shown to be a useful tool in evaluating quality of care from the patients’ perspective in the Netherlands.

**Trial registration:**

SpA-Net is registered in the Netherlands Trial Registry (NTR6740).

**Key Points:**

*• The Commissioning for Quality in Rheumatoid Arthritis Patient-Reported Experience Measure (CQRA-PREM) is a valid measure for assessing patient-centeredness of rheumatology care.*

*• The Dutch version of the CQRA-PREM shows acceptable psychometric properties.*

*• The CQRA-PREM shows to be a useful tool in Plan-Do-Check-Act quality improvement cycles in the Netherlands.*

*• The CQRA-PREM can be used for benchmarking and quality improvement of rheumatology services.*

**Electronic supplementary material:**

The online version of this article (10.1007/s10067-020-05076-6) contains supplementary material, which is available to authorized users.

## Introduction

Evaluating the quality of care provided is helpful to reveal areas of improvement of care, identify best practices and stimulate development and implementation of care innovations. Health care services should also be transparent with respect to care provided, as decision-makers, society and patients have the right to know about the quality of the services available to them [1]. The Institute of Medicine (IOM) identifies six pillars for evaluating quality of care in the current health care system: care that is provided should be safe, effective, patient-centered, timely, efficient, and equitable [2]. Patient-centered care is gaining more attention in the last decade and has become a key part of audits of care organizations [3]. Patient-centered care is defined by the IOM as care that is respectful of, and responsive to, individual patient preferences, needs, and values and ensures that patient values guide all clinical decisions [2]. It is organized around the health needs and expectations of patients rather than around diseases.

There are several advantages of applying patient-centered care in daily practice. A literature overview showed that patient-centered care in patients with rheumatoid arthritis (RA) improved clinical safety and effectiveness [4]. Another study showed that better patient care experiences were associated with higher levels of adherence to treatment [5]. In addition, a patient-centered approach by family physicians and general internists in primary care was associated with decreased utilization of health care services and lower annual medical charges [6]. Therefore, it is important that patients should be asked about their experienced care and improve this where necessary.

Patients’ perspectives on care provided within a certain time period can be evaluated with patient-reported experience measures (PREMs), which focus on aspects of care that matter to patients and thereby identify areas of improvement for health care services. PREMs assess patients’ experiences relating to the structure and/or process of care provided and might include questions relating to outcomes of care provided. PREMs can assess quality of care for the generic population or for a disease-specific population. Disease-specific PREMs are preferred for assessing quality of care provided as generic PREMs might not cover aspects of care that are specific and weighted toward a particular condition [7].

Currently, there are two measures available for assessing patients’ experiences with rheumatic care in the Netherlands: the Consumer Quality Index for patients with Rheumatoid Arthritis (CQ-Index RA) and the Quality of Care Through the Patients’ Eye for all Rheumatic Patients (QUOTE-Rheumatic-Patients) [8, 9]. Both questionnaires are generic measures for assessing the quality of care provided in all health care services available to patients with RA and include the importance patients award to each aspect of quality of care.

However, both questionnaires have several limitations. First, the CQ-Index RA and QUOTE-Rheumatic-Patients contain a large number of questions (115 and 155 questions, respectively), which might be too time-consuming. Second, the CQ-Index RA is disease-specific for RA and therefore not applicable to other rheumatic diseases. Third, both measures are generic for health care services and are not specifically developed for rheumatology services.

In the United Kingdom (UK), a PREM has been developed by the Commissioning for Quality in Rheumatoid Arthritis Patient-Reported Experience Measure (CQRA-PREM) to evaluate patients’ perspectives on care provided in rheumatology units in the UK National Health Service (NHS) [10]. This questionnaire has been developed and validated in RA, modified and validated in other rheumatic conditions, and has been in use since 2015 [10, 11]. The CQRA-PREM includes 23 questions in seven domains aligned to the National Health Service Patient Experience Framework (NPEF) for patient-centered care and one question for evaluating the overall experience of the care provided [10]. The framework of the CQRA-PREM represents the most salient issues in patients’ experiences with hospital care for RA patients and is widely used for assessing patients’ experiences with care provided in several rheumatology units [12–14]. The NPEF domains can be used to identify specific areas of improvement from the patients’ perspective within care departments. The implementation of the CQRA-PREM in rheumatology units was found to be effective in this regard [10].

Currently, a feasible PREM that is applicable to different rheumatic diseases in the setting of the rheumatology unit is lacking in the Netherlands. The primary aim of our study was to test the psychometric properties of the CQRA-PREM by performing qualitative and quantitative analyses in Dutch patients with SpA and RA. A secondary aim was to implement the CQRA-PREM in daily practice in the Netherlands. The results of the questionnaire were evaluated in quality improvement cycles for patient-centeredness of care.

## Methods

### PREM translation

The CQRA-PREM is categorized into seven NPEF domains for patient-centered care (one to five items per domain)*.* Answers are given on a 5-point Likert scale ranging from “strongly disagree” to “strongly agree” (online resources 1 and 2) [15]. Detailed information on the development and validation of the CQRA-PREM can be found elsewhere [10].

The UK version of the CQRA-PREM was independently translated into Dutch by two native Dutch-speaking researchers fluent in English (one rheumatologist and one health care scientist). Discrepancies were discussed, and a consensus version was generated by both forward translators. The consensus version was back-translated by another bilingual Dutch researcher (a methodologist) with no prior knowledge of the questionnaire. A final version was developed by all three translators and was checked by an additional Dutch researcher unfamiliar with the original questionnaire (a rheumatologist).

### Face validity

Face validity of the CQRA-PREM was studied by performing semi-structured focus group interviews with patients. Aspects of patient-centered care that were important to patients and their current experiences with patient-centered care were assessed. A sample of adult patients from the rheumatology outpatient clinic of the Maastricht University Medical Center (MUMC+) was invited to participate. Interviews were planned with approximately five patients per group, until data saturation was reached. Face validity was assessed by comparing aspects of care that were important to patients with domains of the CQRA-PREM.

### Field testing

The psychometric properties of the CQRA-PREM were tested in two ongoing, prospective, disease-specific real-life quality registries in daily practice for patients with SpA and RA in the Netherlands, SpA-Net and DREAM-RA, respectively, in two medical centers (MUMC+ and Medisch Spectrum Twente) in different geographical areas in the Netherlands [16, 17]. Patients in these registries have a clinical diagnosis of SpA or RA and were consecutively included by their rheumatologist. In both registries, outcome measures, results of clinical examinations and laboratory investigations are routinely collected at every outpatient visit through a web-based data collection and quality management application (www.mijnreumacentrum.nl). Outcome measures consist of validated measures of disease activity, physical function and overall health status.

In SpA-Net, disease activity is measured with the Bath Ankylosing Spondylitis Disease Activity Index (BASDAI) and Ankylosing Spondylitis Disease Activity Score (ASDAS) with C-Reactive Protein [18, 19], physical functioning with the Bath Ankylosing Spondylitis Functional Index (BASFI), Health Assessment Questionnaire for Spondyloarthritis (HAQ-S) [20, 21], and disease impact with the Assessment of Spondyloarthritis International Society Health Index score (ASAS HI) [22]. In DREAM-RA, disease activity is measured with the Disease Activity Score for 28 joints with Erythrocyte Sedimentation Rate (DAS28) [23] and physical functioning with the Health Assessment Questionnaire (HAQ) [24]. In both SpA-Net and DREAM-RA, overall health status is assessed with the self-report MOS 36-Item Short Form Health Survey (SF36), resulting in physical component summary (PCS) and mental component summary (MCS) scores [25].

Starting from December 2016, patients from the two medical centers participating in SpA-Net and DREAM-RA were invited to annually complete the CQRA-PREM upon logging in to the application. Questionnaires were only saved if they were fully completed. Patients were informed that individual results are not visible for physicians or nurses. In the current cross-sectional analysis, the most recently completed CQRA-PREM from each patient was included for analyses. Results from outcome measures were included if they were completed within 14 days before or after completing the CQRA-PREM. For measures completed more than once within the 14-day timeframe, the measurement closest in time to the CQRA-PREM administration was selected.

### Implementation and quality improvement

After translation and validation, the CQRA-PREM was implemented in daily practice. Through repeated Plan-Do-Check-Act (PDCA) quality improvement cycles, the results from the CQRA-PREM representing the patients’ perspective on quality of provided care were evaluated at several occasions with rheumatologists and rheumatology nurses from both medical centers. This was followed by group discussions to identify areas for improvement. Several action plans were formulated and executed in clinical practice where possible.

### Statistical analysis

Descriptive statistics were used to describe characteristics of participants of the focus group interviews, of patients who completed the CQRA-PREM in the patient registries, and to describe the relative frequencies of scores in the CQRA-PREM for patients with SpA or RA.

All focus group interviews were audiotaped and transcribed verbatim. In NVIVO V.11, the editing analysis style was used to analyze all transcripts by structurally classifying meaningful quotes into themes and subthemes. Finally, aspects of care that were important for patients were summarized and results were interpreted. Details about the patient inclusion procedure and data analyses have been described elsewhere [17].

In addition to face validity, the following elements of the COSMIN (COnsensus-based Standards for the selection of health status Measurement INstruments) checklist for evaluating the methodological quality of studies on measurement properties were examined: feasibility, interpretability, and internal consistency [26]. Furthermore, homogeneity and divergent validity were also tested.

Feasibility of the CQRA-PREM was determined by calculating the median (interquartile range (IQR)) time patients needed to complete the questionnaire. Interpretability of the CQRA-PREM was evaluated by testing floor and ceiling effects in the average scores of the domains *needs and preferences*, *coordination of care*, *information about care*, *daily living and physical comfort*, and *emotional support*, thereby considering the categorical 5-point Likert scores as linear. Floor and ceiling effects were considered to be present if 15% or more of the patients had the lowest or highest possible average domain score [27].

Internal consistency of a single assessment of the CQRA-PREM was studied within domains containing more than two questions with correlation analyses (Cronbach’s α coefficients) and was considered good if 0.70 ≤ α ≤ 0.95 [28]. Homogeneity within domains containing more than two questions was studied with corrected item-total correlations (*r*_p_) to identify questions with very weak or very strong correlations within the respective domain and was considered good if 0.30 ≤ (*r*_p_) ≤0.70 [29].

Divergent validity was studied through non-parametric Spearman’s rank correlation coefficients (*r*_s_) with average scores of domains and outcome measures for disease activity, daily functioning, health status, and quality of life. Since PREMs are assumed to capture something different than the patient’s condition or outcomes of treatment alone, and in accordance with studies evaluating PREMs in other medical conditions [30], correlations with patient-reported outcomes and clinical outcomes were hypothesized to be weak at most (− 0.30 ≤ *r*_s_ ≤ 0.30), indicating that the measures evaluate relatively distinct constructs [31].

All analyses, except for face validity, were repeated in patients stratified for the use of biologic disease-modifying antirheumatic drugs (bDMARDs) at the time of completing of the CQRA-PREM. Statistical analyses were performed using IBM SPSS Statistics 24.

## Results

The CQRA-PREM was completed by 282 patients with SpA and 376 patients with RA. The average age and the relative number of female patients with SpA were lower compared with patients with RA (52.7 (SD = 12.3) versus 61.5 (SD = 11.9) years and 47.9% versus 64.9% female patients, respectively (Table 1). The median disease duration was 8.6 (min 0.0, max 66.5) years for patients with SpA and 7.7 (min 0.0, max 44.0) years for patients with RA. Use of bDMARDs was 55.0% in patients with SpA and 29.8% in patients with RA. On average, disease activity was high for patients with SpA but low for patients with RA.

**Table 1:** Demographic characteristics and outcome measures of patients in SpA-Net and DREAM-RA

	SpA-Net (*n* = 282)	DREAM-RA (*n* = 376)
Age, years	52.7 (12.3)	61.5 (11.9)
Female, *n* (%)	135 (47.9)	244 (64.9)
Symptom duration, years, median (min-max)	12.9 (0.6–67.5)	NA
Disease duration, years, median (min-max)	8.6 (0.0–66.5)	7.7 (0.0–44.0)
bDMARD use, *n* (%)	155 (55.0%)	112 (29.8%)
Disease activity		
BASDAI [0–10]	4.3 (2.2)	-
ASDAS [0-∞]	2.2 (0.9)	-
DAS28 [0-∞]	-	2.3 (1.2)
Physical function		
HAQ-S [0–3]	0.8 (0.6)	0.8 (0.7)
BASFI [0–10]	3.2 (2.4)	-
ASAS HI [0–19]	5.7 (3.5)	-
Overall health status		
SF36 PCS [0–100]	39.9 (10.4)	40.9 (9.6)
SF36 MCS [0–100]	48.7 (11.2)	50.5 (10.8)

Values expressed as mean (SD), unless otherwise indicated

*bDMARDs* biologic Disease-Modifying Antirheumatic Drugs, *BASDAI* Bath Ankylosing Spondylitis Disease Activity Index, *ASDAS* Ankylosing Spondylitis Disease Activity Score with C-Reactive Protein, *DAS28* Disease Activity Score for 28 joints, *HAQ-S* Health Assessment Questionnaire for Spondyloarthritis, *HAQ* Health Assessment Questionnaire, *BASFI* Bath Ankylosing Spondylitis Functional Index, *ASAS HI* Assessment of Spondyloarthritis international Society Health Index, *SF36* Medical Outcomes Study 36-Question Short Form, *PCS* Physical Component Summary, *MCS* Mental Component Summary, *SpA* Spondyloarthritis, *RA* Rheumatoid Arthritis, *SD* Standard Deviation, *NA* Not Available

Both study populations experienced on average mild difficulties in physical functioning (mean HAQ-S = 0.8 (SpA) and mean HAQ = 0.8 (RA)). The overall health status related to physical health and mental health was comparable in patients with SpA and patients with RA (mean SF36 PCS = 39.9 in SpA and mean SF36 PCS = 40.9 in RA and mean SF36 MCS = 48.7 in SpA and mean SF36 MCS = 50.5 in RA).

The distribution of the scores on the CQRA-PREM was skewed towards positive, indicating that patients have positive experiences with care, and is shown separately for patients with SpA and RA in online resources 1 and 2.

### Face validity

Semi-structured focus group interviews were performed to assess the face validity of the CQRA-PREM. Four focus group interviews were performed with 16 patients (3–5 patients per interview), after which information saturation was reached. Median age of the participants was 62.6 (41–78) years, median symptom duration 17.5 (1–66) years, and 6 (37.5%) were male. The patients identified the following eight aspects as important for providing patient-centered care: (1) *feeling heard by care providers, (2) being involved in shared decision making, (3) being able to visit the same care provider over time, (4) being able to contact care providers when needed, (5) feeling satisfied with the quality of answers, (6) being easily referred to other specialists when needed, (7) having the feeling that there is enough time during appointments, and (8) having appointments on time*. Nearly all these aspects are covered by the CQRA-PREM, except *having appointments on time*.

### Psychometric properties

The median time to complete the CQRA-PREM was 4.7 min (IQR = 2.4) in the total population (4.7 min (IQR = 2.7) in SpA-Net and 4.6 min (IQR = 2.3) in DREAM-RA). Interpretability assessed by floor and ceiling effects in average scores of domains showed ceiling effects (≥ 15%) in the domains *needs and preferences* for both patients with SpA and RA and in the domains *daily living and physical comfort* and *emotional support* for patients with RA (Table 2). Internal consistency of all domains was considered good for patients with SpA or RA, except for the domain *daily living and physical comfort* in patients with RA (α = 0.65) (Table 2). Homogeneity was considered good for each question in the domains *information, education, and self-care* and *daily living and physical comfort* for both patients with SpA and RA (0.3 ≤ (*r*_p_) ≤ 0.7). However, thresholds for homogeneity were exceeded (*r*_p_ > 0.7) by two or more questions within the remaining domains for patients with SpA or RA (Table 2 and online resource 3). The divergent validity showed that, as expected, nearly all domains of the CQRA-PREM were at most weakly correlated with patient-reported outcomes (− 0.30 ≤ *r*_s_ ≤ 0.30) (Table 3).

**Table 2:** Interpretability, internal consistency and homogeneity of the CQRA-PREM in patients with SpA or RA

		Interpretability				Internal consistency (Cronbach’s α)		Homogeneity (*r*_p_) [range]	
PREM domains	*N* questions	SpA-Net		DREAM-RA		SpA-Net	DREAM-RA	SpA-Net	DREAM-RA
		Floor effect	Ceiling effect	Floor effect	Ceiling effect				
1. Needs and preferences	5	0.4%	26.2%	0.0%	27.4%	0.90	0.93	0.69–0.82	0.71–0.85
2. Coordination of care and communication	4	0.4%	11.7%	1.1%	13.6%	0.87	0.91	0.65–0.78	0.71–0.87
3. Information, education, and self-care	4	0.0%	8.5%	0.0%	7.4%	0.72	0.75	0.37–0.63	0.41–0.67
4. Daily living and physical comfort*	2	0.0%	14.9%	0.5%	18.4%	0.70	0.65	0.54	0.48
5. Emotional support*	2	0.0%	13.1%	0.5%	16.5%	0.84	0.91	0.73	0.83
6.Family and friends**	1	NA	NA	NA	NA	NA	NA	NA	NA
7. Access to care**	1	NA	NA	NA	NA	NA	NA	NA	NA

*No corrected item-total correlations range available as domain consists of 2 questions, **No scores available as domain consists of 1 question, *NA* Not Applicable

**Table 3:** Divergent validity of the CQRA-PREM in patients with SpA or RA

	Disease activity			Daily functioning			Disease impact	Overall health status			
	SpA		RA	SpA		RA	SpA	SpA		RA	
Spearman’s rank correlation coefficient (*r*_s_)	BASDAI *n* = 240	ASDAS *n* = 186	DAS28 *n* = 257	BASFI *n* = 200	HAQ-S *n* = 232	HAQ *n* = 342	ASAS HI *n* = 197	SF36 PCS *n* = 260	SF36 MCS *n* = 260	SF36 PCS *n* = 349	SF36 MCS *n* = 349
1. Needs and preferences	− 0.18**	− 0.16*	− 0.08	− 0.12	− 0.10	− 0.05	0.20**	0.12	0.14**	0.00	0.14
2. Coordination of care and communication	− 0.09*	− 0.11	− 0.11	− 0.11	− 0.09	− 0.05	− 0.14*	0.09	0.07	0.10	0.04
3._Information, education, and self-care	− 0.05	− 0.02	− 0.10	− 0.08	− 0.01	− 0.06	− 0.09*	0.06	0.10*	0.07	0.17*
4. Daily living and physical comfort	− 0.48**	− 0.42**	− 0.35**	− 0.34**	− 0.35**	− 0.32**	− 0.44**	0.40**	0.28**	0.34**	0.28**
5. Emotional support	− 0.09*	− 0.11	− 0.25	− 0.20**	− 0.17**	− 0.20*	− 0.18	0.16**	0.10*	0.14	0.14
6. Family and friends	− 0.14*	− 0.18*	0.03	− 0.08	− 0.06	− 0.01	− 0.09	0.03	0.11	−0.05	0.06
7. Access to care	− 0.19**	− 0.25**	− 0.09	− 0.17*	− 0.16*	− 0.10	− 0.22**	0.14*	0.13*	0.09	0.13*
8. Overall experience of care	− 0.21**	− 0.22**	− 0.14	− 0.24*	− 0.19**	− 0.11	− 0.18*	0.17*	0.08	0.13	0.20**

*Spearman’s rank correlation is significant at 0.05 level. **Spearman’s rank correlation is significant at 0.01 level

*BASDAI* Bath Ankylosing Spondylitis Disease Activity Index, *ASDAS* Ankylosing Spondylitis Disease Activity Score, *DAS28* Disease Activity Score for 28 joints, *BASFI* Bath Ankylosing Spondylitis Functional Index, *HAQ-S* Health Assessment Questionnaire for Spondyloarthritis, *HAQ* Health Assessment Questionnaire, *ASAS HI* Assessment of Spondyloarthritis international Society Health Index, *SF36* Medical Outcomes Study 36-Question Short Form, *PCS* physical component summary, *MCS* mental component summary

Patient demographic characteristics and outcome measures were comparable between patients with or without bDMARD use (online resource 4); however, the median disease duration was higher in bDMARD users compared with non-bDMARD users, both in RA and SpA. The median time to complete the CQRA-PREM did not differ between both subgroups (data not shown).

Scores for interpretability and internal consistency were comparable between bDMARD and non-bDMARD users. However, homogeneity differed between patients with and without bDMARDs in both SpA and RA (online resources 5 and 6). Scores for divergent validity were also comparable between bDMARD and non-bDMARD users, except for the domains *access to care* and *overall experience with care* with all outcome measures in patients with SpA (online resources 7 and 8).

In both patients with RA and SpA without bDMARD use, the correlations between these two domains and all outcome measures were, in general, higher compared with patients with bDMARD use, however, at most weakly correlated.

### Implementation

In the PDCA cycles, the CQRA-PREM identified the domain *information, education, and self-care* as an important area of improvement for patients with SpA and RA. Several adjustments in care were made to improve this domain. For example, every new patient with SpA or RA now receives a business card with contact information from his/her treating rheumatologist and is referred to a rheumatology nurse for education. The rheumatology nurse brings under attention the possibility of following a self-management course and supports the patient who is starting a disease-modifying antirheumatic drug. In addition, the awareness about patient organizations, patient groups and self-management programs was further increased by providing leaflets and projecting information on screens in the waiting room.

## Discussion

In this study, we demonstrated that the CQRA-PREM has valid psychometric properties in patients with SpA and RA in clinical practice. In addition, we showed that the CQRA-PREM is a useful tool for assessing the patient-centeredness of care provided and that it is able to identify areas of improvement in a Dutch rheumatology setting.

The CQRA-PREM showed good face validity as aspects that were rated as important to Dutch patients were similar to those raised by patients in the UK. The CQRA-PREM also covers all indicators from the Dutch QUOTE-Rheumatic-Patients [9]. However, the CQ-Index RA includes one domain specifically related to experiences with provided information about medication, which is missing in the CQRA-PREM. On the other hand, the CQRA-PREM includes two domains, *friends and family* and *information, education, and self-care,* that are not addressed in both the CQ-Index RA and QUOTE-Rheumatic-Patients.

Feasibility of the CQRA-PREM was considered acceptable with a median completion time of 4.7 minutes. The homogeneity of the CQRA-PREM showed exceeded thresholds in three domains. This suggests that some questions are redundant within domains. However, our results for internal consistency did not differ from the original development study, both in RA patients (α = 0.65 to α = 0.93 (DREAM-RA) versus α = 0.61 to α = 0.90) and in SpA patients (α = 0.70 to α = 0.90 (SpA-Net) versus α = 0.76 to α = 0.91) [10]. Interpretability of the CQRA-PREM showed ceiling effects for the domains *needs and preferences*, *daily living and physical comfort*, and *emotional support*, which implies that the interpretability of the CQRA-PREM is not valid enough. However, these results could reflect true patients’ experiences, and thus satisfaction with care provided or social desirability bias could have occurred, despite the fact that patients were made aware that results were not visible for physicians and nurses [29]. Moreover, ceiling effects are common in patient experience measures, in contrast with scores for outcome measures [32].

Subgroup analyses were performed in patients with or without bDMARD use at the time of completing the PREM, as hypothetically these patients might have different experiences with provided care. Our study showed that results for feasibility, interpretability, internal consistency, and nearly all analyses for divergent validity were comparable between these groups. Despite the fact that scores for homogeneity differed slightly between these groups, the overall validity of the CQRA-PREM was acceptable in both subgroups.

The Dutch version of the CQRA-PREM was field-tested in two patient registries, and therefore patients might provide higher scores for question 2d (*I feel that the people I see at the clinic are fully up to date with my current situation*), which could result in selection bias. However, it is expected that this has a minimal effect on the validity of the domain *information, education, and self-care*, because it is not solely related to this information but also to having received information on time and receiving enough information to make decisions*.* Results for the domain *coordination and communication* are not biased by field testing in the patient registers, because patients cannot directly contact their care providers through our registries as there is no email functionality in the system. We therefore believe that field testing in patient registries has resulted in only limited selection bias and that the CQRA-PREM can also be used in patients in standard care.

Evaluating the quality of care with PREMs, in addition to Patient-Reported Outcome Measures (PROMs), is important as they measure different aspects of quality of care. PREMs assess patients’ perspectives on the structure and process of provided care, while PROMs specifically assess patients’ perspectives on the outcomes of provided care. Besides that, patients’ perspectives on the quality of provided care might differ from the perspective of health care professionals. As we did in our study, PREMs can be used by care providers to reflect on their own and their team’s performance, indicate specific areas of improvement at clinical and organizational levels, and can be used to evaluate the impact of introduced changes within organizations. Patients benefit from PREMs as it helps them to choose high quality care providers when results for quality of care are made transparent for the public.

This study has several limitations. First, no cognitive debriefing with native Dutch-speaking patients was performed and no cross-cultural validity was assessed. However, besides the three translators, one additional native speaker was included to check the final Dutch version for linguistic and cultural accuracy. Second, no factor analyses were performed to support that the allocation of questions into domains is similar in the Dutch version as in the original CQRA-PREM. However, all translators agreed that each domain of the NPEF is represented by the allocated questions in the Dutch version of the questionnaire. Third, the psychometric properties test-retest reliability, sensitivity-to-change over time, and convergent validity could not be examined in this study. These aspects could be evaluated in further studies, as well the ability of the CQRA-PREM to discriminate between rheumatology units who have more or less attention for providing patient-centered care. Fourth, we acknowledge that selection bias could have occurred due to the registries’ web-based design as patients with low health literacy and/or computer skills might have been excluded from this study. Fifth, the CQRA-PREM did not include a free text field for additional remarks from patients who wanted to elaborate on their results or offer possible solutions for aspects that could be improved in their experiences. Although analyzing these additional remarks in a mixed method approach might be time-consuming, it could provide valuable information for the rheumatic care services.

A strength of our study is that the translation and validation of the CQRA-PREM were combined with focus group interviews to test face validity and with implementation of the measure in daily practice with which, through PDCA cycles, areas for improvement were identified and acted upon. A second strength of this study is that the CQRA-PREM was tested in two real-life registries for SpA and RA in the Netherlands. We were able to validate the questionnaire in daily practice and study divergent validity with recorded outcome measures for disease activity, physical functioning, and overall health status.

In conclusion, the CQRA-PREM has acceptable psychometric properties for assessing quality of care provided in daily practice from the perspective of patients with SpA or RA in the Netherlands. Scores for quality of care provided are not substantially affected by outcome measures for disease activity, physical functioning and overall health status. The CQRA-PREM has shown to be a useful tool in PDCA quality improvement cycles and can be used to optimize patient-centered care in rheumatic health care services as recommended by the IOM.

## Electronic supplementary material

ESM 1 (DOCX 16 kb)

ESM 2 (DOCX 16 kb)

ESM 3 (DOCX 14 kb)

ESM 4 (DOCX 14 kb)

ESM 5 (DOCX 13 kb)

ESM 6 (DOCX 14 kb)

ESM 7 (DOCX 15 kb)

ESM 8 (DOCX 14 kb)

## Data Availability

Additional analyses can be performed upon reasonable request. Contact the principle investigator (a.van.tubergen@mumc.nl) for more information.
