# Supplementary material for: Validation and implementation of a patient-reported experience measure for patients with rheumatoid arthritis and spondyloarthritis in the Netherlands
Source: Clin Rheumatol. 2020 Apr 21;39(10):2889–97. doi: 10.1007/s10067-020-05076-6 (PMC7497348; doi:10.1007/s10067-020-05076-6)
Supplement: Supplementary file 1 — (DOCX 16 kb) [file 10067_2020_5076_MOESM1_ESM.docx]

**Online Resource 1** Results of the CQRA-PREM in patients with SpA (n=282)

| **Domain** | **Question** | **Strongly disagree** | **Disagree** | **Neither agree, neither disagree** | **Agree** | **Strongly agree** | **Not applicable** |
| --- | --- | --- | --- | --- | --- | --- | --- |
| 1. Needs and __preferences | a) Whenever I attended a clinic, I felt that I was treated __respectfully as an individual | 0.7% | 2.5% | 6.7% | 47.9% | 42.2% |  |
|  | b) I was involved as much as I wanted to be in decisions __about my treatment and care | 0.4% | 1.8% | 5.0% | 56.4% | 36.5% |  |
|  | c) My personal circumstances and preferences were taken into account when planning and deciding on my treatment and care | 0.4% | 3.2% | 8.9% | 58.9% | 28.7% |  |
|  | d) I was given information in a way that I could understand | 0.4% | 0.4% | 2.1% | 58.9% | 38.3% |  |
|  | e) I was given enough information to help me make __decisions about my treatment | 0.4% | 1.4% | 4.6% | 58.5% | 35.1% |  |
| 2. Coordination and __communication | a) I was made aware that there is a team of health __professionals looking after me | **-** | 1.8% | 12.4% | 55.3% | 20.6% | 9.9% |
|  | b) When I needed help I was able to access different __members of my health team | **-** | 2.5% | 14.5% | 46.8% | 17.7% | 18.4% |
|  | c) There is a member of my health team who can help me to __see other specialists in the team if I need to | **-** | 3.2% | 20.6% | 42.9% | 14.9% | 18.8% |
|  | d) I feel that the people I see at the clinic are fully up to date _with my current situation | 0.4% | 6.0% | 20.6% | 57.1% | 16.0% |  |
| 3. Information, __education and __self-care | a) I feel that I was given information at the time I needed it | **-**  **-** | 2.5% | 10.3% | 69.5% | 17.7% |  |
|  | b) I feel that I have a good understanding of the treatments __I am on or being offered | **-** | 1.1% | 3.2% | 67.4% | 28.4% |  |
|  | c. I have been told about patient organizations or groups __that can help me | 2.1% | 14.2% | 32.6% | 38.7% | 12.4% |  |
|  | d) I have been offered an opportunity to attend a self-__management program suitable to my needs | 1.8% | 12.1% | 24.1% | 22.7% | 7.80% | 31.6% |
| 4. Daily living and __physical comfort | a) I feel that my rheumatic condition is being controlled enough to let me get on with my daily life and usual activities | 1.4% | 5.7% | 17.7% | 54.6% | 20.6% |  |
|  | b) If I have had a ‘flare’ (when my symptoms get much __worse), I have been able to get help quickly | 0.4% | 3.5% | 16.7% | 42.2% | 15.2% | 22.0% |
| 5. Emotional __support | a) I feel able to approach a member of my health team to _discuss any worries about my condition and my treatment _or their effect on my life | **-** | 4.3% | 25.2% | 55.7% | 14.9% |  |
|  | b) I feel able to discuss personal or intimate issues about _relationships with my health team if I want to | 1.1% | 3.9% | 29.4% | 50.7% | 14.9% |  |
| 6. Family and friends | a) I feel able to take members of my family to outpatient __appointments to become involved in decisions about my __care if I want to | 0.4% | 0.7% | 11.7% | 51.4% | 35.8% |  |
| 7. Access to care | a) At appointments, I feel that I have enough time with the __health care professional to cover everything I want to __discuss | - | 2.8% | 7.8% | 58.2% | 31.2% |  |
|  | b) I have had clinic appointments cancelled unexpectedly | **Yes**  11.7% | **No**  88.3% |  |  |  |  |
|  | c) If yes, how long have you had to wait for a new __appointment? | **<1 week**  39.4% | **1-3 weeks**  36.4% | **4-6 weeks**  15.2% | **7-12 weeks**  **-** | **>12 weeks**  9.1% |  |
|  | d) I have needed extra treatment or a change of treatment | **Yes**  22.3% | **No**  77.7% |  |  |  |  |
|  | e) If yes, how long did it take for this to happen? | **<1 week**  38.1% | **1-3 weeks**  35.0% | **4-6 weeks**  12.7% | **7-12 weeks**  6.4% | **>12 weeks**  7.9% |  |
| 8. Overall __experienced care | a) Overall in the past year, I have had a good experience of __care for my rheumatoid arthritis | - | 2.1% | 6.7% | 58.2% | 33.0% |  |
