# Supplementary material for: Validation and implementation of a patient-reported experience measure for patients with rheumatoid arthritis and spondyloarthritis in the Netherlands
Source: Clin Rheumatol. 2020 Apr 21;39(10):2889–97. doi: 10.1007/s10067-020-05076-6 (PMC7497348; doi:10.1007/s10067-020-05076-6)
Supplement: Supplementary file 2 — (DOCX 16 kb) [file 10067_2020_5076_MOESM2_ESM.docx]

**Online Resource 2** Results of the CQRA-PREM in patients with RA (n=376)

| **Domain** | **Question** | **Strongly disagree** | **Disagree** | **Neither agree, neither disagree** | **Agree** | **Strongly agree** | **Not applicable** |
| --- | --- | --- | --- | --- | --- | --- | --- |
| 1. Needs and __preferences | a) Whenever I attended a clinic, I felt that I was treated __respectfully as an individual | - | 0.8% | 1.9% | 56.6% | 40.7% |  |
|  | b) I was involved as much as I wanted to be in decisions __about my treatment and care | - | 0.3% | 3.7% | 61.4% | 34.6% |  |
|  | c) My personal circumstances and preferences were taken into account when planning and _deciding on my treatment and care | - | 0.3% | 3.7% | 63.0% | 32.7% |  |
|  | d) I was given information in a way that I could understand | - | 0.5% | 1.6% | 60.4% | 37.5% |  |
|  | e) I was given enough information to help me make __decisions about my treatment | - | 0.3% | 2.9% | 63.0% | 33.8% |  |
| 2. Coordination and __communication | a) I was made aware that there is a team of health __professionals looking after me | 0.5% | 1.3% | 7.6% | 57.7% | 21.0% | 12.8% |
|  | b) When I needed help I was able to access different __members of my health team | 0.5% | 1.3% | 7.7% | 55.3% | 16.8% | 18.4% |
|  | c) There is a member of my health team who can help me to __see other specialists in the team if I need to | 0.5% | 1.3% | 13.0% | 45.5% | 16.0% | 23.7% |
|  | d) I feel that the people I see at the clinic are fully up to date _with my current situation | 1.3% | 3.2% | 13.3% | 63.8% | 18.4% |  |
| 3. Information, __education and __self-care | a) I feel that I was given information at the time I needed it | -  - | 1.1% | 6.6% | 72.6% | 19.7% |  |
|  | b) I feel that I have a good understanding of the treatments __I am on or being offered | -  - | 0.3% | 4.0% | 67.6% | 28.2% |  |
|  | c. I have been told about patient organizations or groups __that can help me | 1.6% | 8.8% | 28.2% | 51.3% | 10.1% |  |
|  | d) I have been offered an opportunity to attend a self-__management program suitable to my needs | 0.8% | 10.9% | 22.1% | 27.4% | 5.3% | 33.5% |
| 4. Daily living and __physical comfort | a) I feel that my rheumatic condition is being controlled enough to let me get on with my daily life and usual activities | 0.8% | 3.2% | 12.5% | 59.8% | 23.7% |  |
|  | b) If I have had a ‘flare’ (when my symptoms get much __worse), I have been able to get help quickly | 0.5% | 1.1% | 13.8% | 44.1% | 18.9% | 21.5% |
| 5. Emotional __support | a) I feel able to approach a member of my health team to _discuss any worries about my condition and my treatment _or their effect on my life | 0.% | 1.90% | 18.4% | 60.1% | 18.9% |  |
|  | b) I feel able to discuss personal or intimate issues about _relationships with my health team if I want to | 1.1% | 2.4% | 18.9% | 60.1% | 17.6% |  |
| 6. Family and friends | a) I feel able to take members of my family to outpatient __appointments to become involved in decisions about my __care if I want to | 0.3% | 0.5% | 5.3% | 63.6% | 30.3% |  |
| 7. Access to care | a) At appointments, I feel that I have enough time with the __health care professional to cover everything I want to __discuss | - | 0.3% | 4.3% | 64.4% | 31.1% |  |
|  | b) I have had clinic appointments cancelled unexpectedly | **Yes**  7.4% | **No**  92.6% |  |  |  |  |
|  | c) If yes, how long have you had to wait for a new __appointment? | **<1 week**  32.1% | **1-3 weeks**  60.7% | **4-6 weeks**  **-** | **7-12 weeks**  7.1% | **>12 weeks**  **-** |  |
|  | d) I have needed extra treatment or a change of treatment | **Yes**  22.1% | **No**  77.9% |  |  |  |  |
|  | e) If yes, how long did it take for this to happen? | **<1 week**  67.5% | **1-3 weeks**  28.9% | **4-6 weeks**  1.2% | **7-12 weeks**  2.4% | **>12 weeks**  **-** |  |
| 8. Overall __experienced care | a) Overall in the past year, I have had a good experience of __care for my rheumatoid arthritis | 0.3% | 0.5% | 5.3% | 59.8% | 34.0% |  |
