# Supplementary material for: Validation and implementation of a patient-reported experience measure for patients with rheumatoid arthritis and spondyloarthritis in the Netherlands
Source: Clin Rheumatol. 2020 Apr 21;39(10):2889–97. doi: 10.1007/s10067-020-05076-6 (PMC7497348; doi:10.1007/s10067-020-05076-6)
Supplement: Supplementary file 3 — (DOCX 14 kb) [file 10067_2020_5076_MOESM3_ESM.docx]

**Online Resource 3** Homogeneity of the CQRA-PREM in patients with SpA and RA

| **Domains CQRA-PREM** |  | **Homogeneity**  **(r_p_)** | |
| --- | --- | --- | --- |
|  |  | **SpA-Net** | **DREAM-RA** |
| 1. Needs and preferences | a) Whenever I attended a clinic, I felt that I was treated respectfully as an individual | 0.69 | 0.71 |
|  | b) I was involved as much as I wanted to be in decisions about my treatment and care | 0.79 | 0.84 |
|  | c) My personal circumstances (see note 1 below) and preferences were taken into __account when planning and deciding on my treatment and care | 0.69 | 0.82 |
|  | d) I was given information in a way that I could understand | 0.81 | 0.82 |
|  | e) I was given enough information to help me make decisions about my treatment | 0.82 | 0.85 |
| 2. Coordination and communication | a) I was made aware that there is a team of health professionals looking after me | 0.78 | 0.77 |
|  | b) When I needed help I was able to access different members of my health team | 0.78 | 0.87 |
|  | c) There is a member of my health team who can help me to see other specialists in the __team if I need to | 0.73 | 0.81 |
|  | d) I feel that the people I see at the clinic are fully up to date with my current situation | 0.65 | 0.71 |
| 3. Information, education and self-care | a) I feel that I was given information at the time I needed it | 0.50 | 0.50 |
|  | b) I feel that I have a good understanding of the treatments I am on or being offered | 0.37 | 0.41 |
|  | c. I have been told about patient organizations or groups that can help me | 0.60 | 0.66 |
|  | d) I have been offered an opportunity to attend a self-management program suitable to __my needs | 0.63 | 0.67 |
| 4. Daily living and physical comfort | a) I feel that my rheumatic condition is being controlled enough to let me get on with my __daily life and usual activities | 0.54 | 0.49 |
|  | b) If I have had a ‘flare’ (when my symptoms get much worse), I have been able to get __help quickly |  |  |
| 5. Emotional support | a) I feel able to approach a member of my health team to discuss any worries about my __condition and my treatment or their effect on my life | 0.73 | 0.83 |
|  | b) I feel able to discuss personal or intimate issues about relationships with my health __team if I want to |  |  |
