# Supplementary material for: Validation and implementation of a patient-reported experience measure for patients with rheumatoid arthritis and spondyloarthritis in the Netherlands
Source: Clin Rheumatol. 2020 Apr 21;39(10):2889–97. doi: 10.1007/s10067-020-05076-6 (PMC7497348; doi:10.1007/s10067-020-05076-6)
Supplement: Supplementary file 4 — (DOCX 14 kb) [file 10067_2020_5076_MOESM4_ESM.docx]

Online resource 4 Demographic characteristics and outcomes measures of patients in SpA-Net and DREAM-RA stratified for bDMARD use

|  | **SpA-Net**  (n=282) | | **DREAM-RA**  (n=376) | |
| --- | --- | --- | --- | --- |
|  | **Non-bDMARD use**  n = 127 | **bDMARD**  **use**  n = 155 | **Non-bDMARD use**  n =264 | **bDMARD**  **use**  n =112 |
| **Age, years** | 55.1 (12.5) | 50.7 (11.8) | 59.6 (12.5) | 59.3 (10.2) |
| **Female, n (%)** | 60 (47.2%) | 75 (48.4%) | 165 (62.5%) | 79 (70.5%) |
| **Symptom duration in years, median (min-max)** | 12.3 (0.7 – 67.5) | 15.2 (0.6 – 52.9) | NA | NA |
| **Disease duration in years, median (min-max)** | 7.5 (0.0 – 66.5) | 10.1 (0.2 – 52.9) | 5.2 (0.0 – 39.2) | 14.6 (0.1 – 44.0) |
| **Disease activity**  BASDAI [0-10]  ASDAS [0-∞]  DAS28 [0-∞] | 4.3 (2.1)  2.2 (0.9)  - | 4.4 (2.3)  2.2 (0.9)  - | -  -  2.2 (1.1) | -  -  2.5 (1.1) |
| **Physical function**  HAQ(-S) [0-3]  BASFI [0-10]  ASAS-HI [0-19] | 0.7 (0.6)  2.9 (2.3)  5.2 (3.4) | 0.8 (0.6)  3.4 (2.4)  6.2 (3.4) | 0.7 (0.6)  -  - | 1.0 (0.7) |
| **Overall health status**  SF36 PCS [0-100]  SF36 MCS [0-100] | 41.0 (9.9)  49.4(10.3) | 39.0 (10.6)  48.2 (11.8) | 41.8 (9.7)  50.7 (10.9) | 39.0 (9.0)  50.0 (10.8) |
| Values expressed as mean (SD), unless otherwise indicated.  bDMARDs = biologic Disease-Modifying Antirheumatic Drugs, BASDAI = Bath Ankylosing Spondylitis Disease Activity Index, ASDAS = Ankylosing Spondylitis Disease Activity Score C-Reactive Protein, DAS28 = Disease Activity Score for 28 joints, HAQ-S = Health Assessment Questionnaire for Spondyloarthritis, HAQ = Health Assessment Questionnaire, BASFI = Bath Ankylosing Spondylitis Functional Index, ASAS-HI = Assessment of SpondyloArthritis international Society Health Index SF36 = Medical Outcomes Study 36-Question Short Form, PCS = Physical Component Summary, MCS = Mental Component Summary, SpA = spondyloarthritis, RA = rheumatoid arthritis, SD = standard deviation, NA = not available | | | | |
