# Supplementary material for: Validation and implementation of a patient-reported experience measure for patients with rheumatoid arthritis and spondyloarthritis in the Netherlands
Source: Clin Rheumatol. 2020 Apr 21;39(10):2889–97. doi: 10.1007/s10067-020-05076-6 (PMC7497348; doi:10.1007/s10067-020-05076-6)
Supplement: Supplementary file 5 — (DOCX 13 kb) [file 10067_2020_5076_MOESM5_ESM.docx]

Online resource 5 Subgroup analyses in patients with SpA stratified for bDMARD use: Interpretability, internal consistency and homogeneity

|  |  | **Interpretability** | | | | **Internal consistency**  **(Cronbach’s α)** | | **Homogeneity**  **(r_p_) [range]** | |
| --- | --- | --- | --- | --- | --- | --- | --- | --- | --- |
| **PREM domains** | **N questions** | **Non-bDMARD**  **use**  n = 127 | | **bDMARD use**  n = 155 | | **Non-bDMARD use**  n =127 | **bDMARD use**  n = 155 | **Non-bDMARD use**  n = 127 | **bDMARD use**  n = 155 |
|  |  | **Floor effect** | **Ceiling effect** | **Floor effect** | **Ceiling effect** |  |  |  |  |
| 1. Needs and preferences | 5 | 0.8% | 26.8% | 0.0% | 25.8% | 0.93 | 0.85 | 0.83-0.87 | 0.55-0.77 |
| 2. Coordination of care and communication | 4 | 0.0% | 14.2% | 0.6% | 9.7% | 0.90 | 0.86 | 0.66-0.84 | 0.65-0.75 |
| 3. Information, education and self-care | 4 | 0.0% | 7.9% | 0.0% | 9.0% | 0.72 | 0.71 | 0.49-0.56 | 0.24-0.71 |
| 4. Daily living and physical comfort* | 2 | 0.0% | 15.0% | 0.0% | 14.8% | 0.75 | 0.65 | 0.61 | 0.48 |
| 5. Emotional support* | 2 | 0.0% | 15.0% | 0.0% | 11.6% | 0.86 | 0.83 | 0.75 | 0.71 |
| 6.Family and friends** | 1 | NA | NA | NA | NA | NA | NA | NA | NA |
| 7. Access to care** | 1 | NA | NA | NA | NA | NA | NA | NA | NA |
| * No corrected item-total correlations range available as domain consists of 2 questions, ** No scores available as domain consists of 1 question,  NA = Not Applicable, bDMARDs = biologic Disease-Modifying Antirheumatic Drugs | | | | | | | | | |
