# Supplementary material for: Validation and implementation of a patient-reported experience measure for patients with rheumatoid arthritis and spondyloarthritis in the Netherlands
Source: Clin Rheumatol. 2020 Apr 21;39(10):2889–97. doi: 10.1007/s10067-020-05076-6 (PMC7497348; doi:10.1007/s10067-020-05076-6)
Supplement: Supplementary file 6 — (DOCX 14 kb) [file 10067_2020_5076_MOESM6_ESM.docx]

Online resource 6 Subgroup analyses in patients with RA stratified for bDMARD use: Interpretability, internal consistency and homogeneity

|  |  | **Interpretability** | | | | **Internal consistency**  **(Cronbach’s α)** | | **Homogeneity**  **(r_p_ ) [range]** | |
| --- | --- | --- | --- | --- | --- | --- | --- | --- | --- |
| **PREM domains** | **N questions** | **Non-bDMARD**  **use**  n = 264 | | **bDMARD**  **use**  n = 112 | | **Non-bDMARD**  **use**  n = 264 | **bDMARD**  **use**  n = 112 | **Non-bDMARD**  **use**  n = 264 | **bDMARD**  **use**  n = 112 |
|  |  | **Floor effect** | **Ceiling effect** | **Floor effect** | **Ceiling effect** |  |  |  |  |
| 1. Needs and preferences | 5 | 0.0% | 23.5% | 0.0% | 36.6% | 0.92 | 0.94 | 0.69 – 0.84 | 0.74 – 0.87 |
| 2. Coordination of care and communication | 4 | 1.5% | 12.9% | 0.0% | 15.2% | 0.92 | 0.84 | 0.75 – 0.90 | 0.59 – 0.78 |
| 3. Information, education and self-care | 4 | 0.0% | 8.0% | 0.0% | 6.3% | 0.77 | 0.67 | 0.45 - 0.69 | 0.26 – 0.64 |
| 4. Daily living and physical comfort* | 2 | 0.8% | 19.3% | 0.0% | 16.1% | 0.64 | 0.67 | 0.48 | 0.50 |
| 5. Emotional support* | 2 | 0.4% | 16.7% | 0.9% | 16.1% | 0.90 | 0.92 | 0.82 | 0.85 |
| 6.Family and friends** | 1 | NA | NA | NA | NA | NA | NA | NA | NA |
| 7. Access to care** | 1 | NA | NA | NA | NA | NA | NA | NA | NA |
| * No corrected item-total correlations range available as domain consists of 2 questions, ** No scores available as domain consists of 1 question,  NA = Not Applicable, bDMARDs = biologic Disease-Modifying Antirheumatic Drugs | | | | | | | | | |
