# Supplementary material for: Validation and implementation of a patient-reported experience measure for patients with rheumatoid arthritis and spondyloarthritis in the Netherlands
Source: Clin Rheumatol. 2020 Apr 21;39(10):2889–97. doi: 10.1007/s10067-020-05076-6 (PMC7497348; doi:10.1007/s10067-020-05076-6)
Supplement: Supplementary file 7 — (DOCX 15 kb) [file 10067_2020_5076_MOESM7_ESM.docx]

**Online resource 7** Subgroup analyses in patients with SpA stratified for bDMARD use: Divergent validity

|  | **Disease activity** | | | | **Daily functioning** | | | | | **Overall health status** | | | **Generic health-related quality of life** | | | | |
| --- | --- | --- | --- | --- | --- | --- | --- | --- | --- | --- | --- | --- | --- | --- | --- | --- | --- |
|  | **BASDAI**  n = 240 | | **ASDAS**  n = 186 | | **BASFI**  n = 200 | | **HAQ-S**  n = 232 | | | **ASAS HI**  n = 197 | | | **SF36 PCS**  n = 260 | | | **SF36 MCS**  n = 260 | |
| **Spearman’s correlation**  **coefficient (r_s_)** | **Non-bDMARD use**  n = 103 | **bDMARD use**  n = 137 | **Non-bDMARD use**  n = 80 | **bDMARD use**  n = 106 | **Non-bDMARD use**  n = 90 | **bDMARD use**  n = 110 | **Non-bDMARD use**  n = 100 | **bDMARD use**  n = 132 | **Non-bDMARD use**  n = 88 | | **bDMARD use**  n = 109 | **Non-bDMARD use**  n = 112 | | **bDMARD use**  n = 148 | **Non-bDMARD use**  n = 112 | | **bDMARD use**  n = 148 |
| 1. Needs and preferences | -0.29** | -0.01 | -0.28* | -0.07 | -0.20 | -0.07 | -0.18 | -0.07 | -0.31 | | 0.13 | 0.15 | | 0.14 | 0.16 | | 0.13 |
| 2. Coordination of care and _communication | -0.15 | -0.04 | -0.16 | -0.07 | -0.21 | -0.03 | -0.16 | -0.04 | -0.24* | | -0.07 | 0.19 | | 0.05 | 0.06 | | 0.07 |
| 3. Information, education __and self-care | -0.15 | 0.03 | -0.13 | 0.05 | -0.20 | 0.01 | -0.14 | -0.09 | -0.16 | | -0.06 | 0.18 | | 0.02 | 0.09 | | 0.12 |
| 4.Daily living and physical _comfort | -0.46** | -0.49** | -0.52** | -0.36** | -0.45** | -0.27** | -0.42** | -0.32** | -0.47** | | -0.44** | 0.46** | | 0.38** | 0.16 | | 0.36* |
| 5. Emotional support | -0.14 | -0.06 | -0.14 | -0.09 | -0.26 | -0.15 | -0.25* | -0.14 | -0.28** | | -0.12 | 0.28 | | 0.09 | 0.14 | | 0.07 |
| 6. Family and friends | -0.29** | -0.05 | -0.30** | -0.10 | -0.03 | -0.14 | -0.16 | -0.02 | -0.14 | | -0.08 | 0.04 | | 0.06 | 0.13 | | 0.09 |
| 7. Access to care | -0.39** | -0.04 | -0.43** | -0.12 | -0.35** | -0.02 | -0.31** | -0.07 | -0.33** | | -0.14 | 0.27 | | 0.05 | 0.16 | | 0.10 |
| 8. Overall experience of care | -0.38** | -0.07 | -0.40 | -0.09 | -0.41** | -0.09 | -0.32** | -0.10 | -0.42** | | 0.03 | 0.32** | | 0.08 | 0.16 | | 0.03 |
| * Spearman rank correlation is significant at 0.05 level ** Spearman rank correlation is significant at 0.01 level  bDMARDs = biologic Disease-Modifying Antirheumatic Drugs, BASDAI = Bath Ankylosing Spondylitis Disease Activity Index, ASDAS = Ankylosing Spondylitis Disease Activity Score, BASFI = Bath Ankylosing Spondylitis Functional Index, HAQ-S = Health Assessment Questionnaire for Spondyloarthritis, ASAS-HI = Assessment of SpondyloArthritis international Society Health Index, SF36 = Medical Outcomes Study 36-Question Short Form, PCS = Physical Component Summary, MCS = Mental Component Summary | | | | | | | | | | | | | | | | | |
