# Supplementary material for: Validation and implementation of a patient-reported experience measure for patients with rheumatoid arthritis and spondyloarthritis in the Netherlands
Source: Clin Rheumatol. 2020 Apr 21;39(10):2889–97. doi: 10.1007/s10067-020-05076-6 (PMC7497348; doi:10.1007/s10067-020-05076-6)
Supplement: Supplementary file 8 — (DOCX 14 kb) [file 10067_2020_5076_MOESM8_ESM.docx]

**Online resource 8** Subgroup analyses in patients with RA stratified for bDMARD use: Divergent validity

|  | **Disease activity** | | **Daily functioning** | | **Generic health-related quality of life** | | | |
| --- | --- | --- | --- | --- | --- | --- | --- | --- |
|  | **DAS28**  n = 257 | | **HAQ**  n = 342 | | **SF36 PCS**  n = 349 | | **SF36 MCS**  n = 349 | |
| **Spearman’s correlation**  **coefficient (r_s_)** | **Non-bDMARD use**  n = 180 | **bDMARD**  **use**  n = 77 | **Non-bDMARD use**  n = 238 | **bDMARD**  **use**  n = 104 | **Non-bDMARD use**  n = 240 | **bDMARD**  **use**  n = 109 | **Non-bDMARD use**  n = 240 | **bDMARD**  **use**  n = 109 |
| 1._Needs and preferences | -0.09 | -0.11 | -0.12 | -0.04 | 0.00 | 0.06 | 0.08 | 0.30** |
| 2._ Coordination of care and communication | -0.15* | 0.00 | -0.09 | -0.03 | 0.13* | 0.07 | -0.01 | 0.20* |
| 3._ Information, education and self-care | -0.14 | 0.03 | -0.11 | -0.04 | 0.09 | 0.07 | 0.17* | 0.20* |
| 4.Daily living and physical comfort | -0.43** | -0.14 | 0.35** | -0.37** | 0.38** | 0.30** | 0.28** | 0.30** |
| 5. Emotional support | -0.28** | -0.18 | -0.26** | -0.16 | 0.18** | 0.06 | 0.12 | 0.20* |
| 6. Family and friends | 0.02 | 0.05 | -0.09 | 0.10 | 0.02 | -0.21 | 0.04 | 0.09 |
| 7. Access to care | -0.13 | 0.03 | -0.13* | -0.06 | 0.12 | -0.01 | 0.04 | 0.34** |
| 8. Overall experience of care | -0.23** | 0.06 | -0.18** | -0.08 | 0.18** | 0.06 | 0.15* | 0.31** |
| * Spearman rank correlation is significant at 0.05 level ** Spearman rank correlation is significant at 0.01 level  bDMARDs = biologic Disease-Modifying Antirheumatic Drugs, DAS28 = Disease activity score for 28 joints, HAQ = Health Assessment Questionnaire, SF36 = Medical Outcomes Study 36-Question Short Form, PCS = Physical Component Summary, MCS = Mental Component Summary | | | | | | | | |
